# Immunogenicity of a *Plasmodium vivax* vaccine based on the duffy binding protein formulated using adjuvants compatible for use in humans

**DOI:** 10.1038/s41598-023-40043-6

**Published:** 2023-08-25

**Authors:** Francisco J. Martinez, Micheline Guillotte-Blisnick, Christèle Huon, Patrick England, Jean Popovici, Hélène Laude, Laurence Arowas, Marie-Noëlle Ungeheuer, Jenny M. Reimer, Darrick Carter, Steve Reed, Paushali Mukherjee, Virander S. Chauhan, Chetan E. Chitnis

**Affiliations:** 1 Unité de Biologie de Plasmodium et Vaccins, Institut Pasteur, Université Paris Cité, 25-28 Rue du Dr. Roux, 75015 Paris, France; 2 Plate-Forme de Biophysique Moléculaire, CNRS UMR 3528, Institut Pasteur, Université Paris Cité, Paris, France; 3 Malaria Research Unit, Institut Pasteur du Cambodge, Phnom Penh, Cambodia; 4 Investigational Clinical Service and Access to Research Bio-Resources (ICAReB), Institut Pasteur, Paris, France; 5 Novavax AB, Kungsgatan 109, 753 18 Uppsala, Sweden; 6 HDT Bio, Seattle, WA USA; 7 PAI Life Sciences Inc., Seattle, WA USA; 8 Multi-Vaccines Development Program, ICGEB Campus, New Delhi, India; 9 International Centre for Genetic Engineering and Biotechnology (ICGEB), New Delhi, India

**Keywords:** Parasitology, Vaccines, Immunology, Microbiology

## Abstract

The invasion of reticulocytes by *Plasmodium vivax* merozoites is dependent on the interaction of the *Plasmodium vivax* Duffy Binding Protein (PvDBP) with the Duffy antigen receptor for chemokines (DARC). The N-terminal cysteine-rich region II of PvDBP (PvDBPII), which binds DARC, is a leading *P. vivax* malaria vaccine candidate. Here, we have evaluated the immunogenicity of recombinant PvDBPII formulated with the adjuvants Matrix-M and GLA-SE in mice. Analysis of the antibody responses revealed comparable ELISA recognition titres as well as similar recognition of native PvDBP in *P. vivax* schizonts by immunofluorescence assay. Moreover, antibodies elicited by the two adjuvant formulations had similar functional properties such as avidity, isotype profile and inhibition of PvDBPII-DARC binding. Furthermore, the anti-PvDBPII antibodies were able to block the interaction of DARC with the homologous PvDBPII SalI allele as well as the heterologous PvDBPII PvW1 allele from a Thai clinical isolate that is used for controlled human malaria infections (CHMI). The cross-reactivity of these antibodies with PvW1 suggest that immunization with the PvDBPII SalI strain should neutralize reticulocyte invasion by the challenge *P. vivax* strain PvW1.

## Introduction

Of the five *Plasmodium* species that infect humans, *P. falciparum* and *P. vivax* are the most predominant. While *P. falciparum* is the most virulent, *P. vivax* has the widest geographic distribution across the world^1^. Prevention and control measures have resulted in a substantial decrease of malaria cases and malaria-related mortality over the past two decades^2^. However, the control measures were found to be more effective for *P. falciparum* than *P. vivax*, due to the unique biology of *P. vivax*^3^. The early appearance of *P. vivax* gametocytes leads to rapid transmission even before the first clinical symptoms appear and the patient seeks treatment. In addition, the latent hypnozoite stage in the liver—which is undetectable—can cause blood stage infection weeks, months or years after the initial infection. New tools are needed if we want to achieve elimination of *P. vivax.* An effective vaccine can play an important role in achieving this goal.

The clinical symptoms of malaria are entirely attributed to the blood stage of the *P. vivax* life cycle, which involves infection of reticulocytes, intracellular replication, and egress of next generation merozoites that go on to invade fresh reticulocytes. As the invasion and replication cycle progresses, parasitemia rises leading to clinical symptoms as the fever threshold is crossed. *P. vivax* invasion of reticulocytes is a complex process that involves multiple receptor-ligand interactions. A key interaction that appears to be essential for invasion is mediated by the interaction of the *P. vivax* Duffy binding protein (PvDBP) with the Duffy antigen receptor for chemokines (DARC) on reticulocytes^4,5^. The binding domain of PvDBP was mapped to a 39 kDa conserved cysteine-rich region, referred to as region II (PvDBPII)^6^. Upon natural exposure, binding inhibitory antibody responses elicited against PvDBPII have been shown to correlate with protection against *P. vivax* infection^7,8^. In addition, it was found that these high-titre anti-PvDBPII binding inhibitory antibodies can block DARC binding by diverse PvDBPII variants.

PvDBPII based on the reference Salvador I (SalI) sequence has been expressed as a recombinant vaccine antigen in *E. coli* and purified to homogeneity in its native conformation^9–12^. Recombinant PvDBPII formulated with diverse adjuvants was tested in animal models^10,12,13^. In these studies, PvDBPII formulated with glucosylpyranosyl lipid adjuvant-stable emulsion (GLA-SE) was found to elicit high titre anti-PvDBPII binding inhibitory antibodies that were strain-transcending and blocked receptor-binding by diverse PvDBPII polymorphic domains. These observations from pre-clinical and field studies provide the rationale for the development of a vaccine based on PvDBPII that could protect against diverse strains of *P. vivax*. PvDBPII/GLA-SE was subsequently tested in a dose-escalation Phase I clinical trial, which demonstrated that it was safe and immunogenic eliciting high titre binding inhibitory antibodies against PvDBPII^14^.

At this time, other adjuvants such as the saponin-based adjuvant Matrix-M extracted from *Quillaja saponaria* (QS)^15^ have become available and were being used for formulation of experimental recombinant protein-based vaccines including the *P. falciparum* vaccine R21 based on the circumsporozoite protein (PfCSP)^16^. R21 formulated with Matrix-M demonstrated excellent safety and immunogenicity profiles^17^. In addition, a PvCSP-based vaccine, Rv21, adjuvanted with Matrix-M showed protection in mice in pre-clinical studies^18^. Given the potential of combining PvDBPII with a PvCSP-based vaccine such as Rv21, it was decided to evaluate the immunogenicity of PvDBPII formulated with Matrix-M. Here, we test the immunogenicity of PvDBPII formulated with Matrix-M and GLA-SE in mice. PvDBPII formulated with both Matrix-M or GLA-SE elicits similar levels of high-titre PvDBPII-specific antibodies that block receptor binding to the homologous PvDBPII variant SalI. Antibodies elicited by PvDBPII SalI were also tested for inhibition of receptor-binding by PvDBPII derived from the Thai *P. vivax* clinical isolate PvW1^19^, which has been developed as a blood-stage challenge strain to evaluate efficacy of vaccines in controlled human malaria infections (CHMI). Antibodies elicited by recombinant PvDBPII SalI formulated with Matrix-M and GLA-SE inhibited receptor binding by PvDBPII PvW1 with similar efficiency. These observations demonstrated the ability of recombinant PvDBPII formulated with both Matrix-M and GLA-SE to elicit potent cross-reactive antibodies that could potentially protect against infection by the heterologous *P. vivax* challenge strain PvW1.

## Experimental procedures

### Expression of PvDBPII and DARC recombinant proteins

Recombinant PvDBPII variants SalI and PvW1 were produced as previously described^9–12^. Briefly, the PvDBPII codon-optimized gene was cloned into the pET28a (+) vector (including a C-terminal 6xHis tag) and the resultant plasmid was used to transform the *E. coli* strain BLR(DE3). PvDBPII was expressed by fed-batch fermentation process, cells were lysed and PvDBPII solubilized and purified by nickel-charged nitrilotriacetic acid (Ni–NTA) affinity chromatography. PvDBPII was refolded by rapid dilution method, dialyzed, and finally purified by cation exchange (SP Sepharose column) and gel filtration (Superdex 200 column) chromatography. Protein purity was evaluated by sodium dodecyl sulfate–polyacrylamide gel electrophoresis (SDS-PAGE), acrylamide at 12%, and stained with PageBlue Protein Staining solution (Thermo Fisher). His-tagged recombinant PvDBPII was detected by Western blotting using primary mouse anti-His antibody (dilution 1:500, Sigma) and secondary horseradish peroxidase (HRP)-conjugated anti-mouse antibody (dilution 1:2500, Sigma). The correct folding of the recombinant PvDBPII protein was confirmed in functional erythrocyte binding and DARC-binding assays as described below. The final monomeric PvDBPII was stored at − 80 °C.

Two plasmids encoding N-terminal DARC sequence (nDARC) (first 60 codons of human DARC (FyB) with the tyrosine (Y) or phenylalanine (F) in position 41) fused to the Fc region of human IgG1 in the mammalian expression vector pCDM8 were used for expression in mammalian HEK293T cells^20^. The plasmid containing the human tyrosylprotein sulfotransferase-2 (TPST-2) was co-transfected with the DARC plasmids to ensure sulfation of recombinant DARC. Recombinant nDARC(Y)-Fc or nDARC(F)-Fc was purified from culture supernatants by protein G affinity chromatography and stored at − 80 °C.

### Production of antisera against PvDBPII formulated with Matrix-M and GLA-SE in mice

Two groups of five BALB/c mice each were immunized with 15 μg of PvDBPII variant SalI formulated either with 5 μg of Matrix-M or 1 μg of GLA-SE with a priming dose at day 0 and two boosts at days 28 and 56. This study was carried out in accordance with the recommendations in the Guide for the Care and Use of Laboratory Animals of the Institut Pasteur and the ARRIVE guidelines. Serum samples were collected at day − 1, 27, 55 and 70. ELISA protocol described below was performed on samples from days 0, 27, 55 and 70. Binding Inhibition assay, Avidity, Isotyping and Immunofluorescence assay (IFA) were performed only from day 70 serum samples.

### Erythrocyte binding assay

Human red blood cells (RBCs) were obtained from duffy positive (Fy+) and duffy negative (Fy−) donors. An informed written consent was obtained from patients prior to enrollment. All procedures were carried out in strict accordance with relevant guidelines and regulations. For duffy phenotyping, 2 ml of whole blood were analyzed using immunological hemagglutination. Subjects were considered as duffy negative when their red blood cells harbored neither FyA or FyB antigens whereas subjects were considered as duffy positive when their red blood cells harbored FyA or FyB or both antigens. RBCs (1 × 10^8^) were incubated with 1 μg of PvDBPII SalI or PvW1 in 100 μl of Roswell Park Memorial Institute (RPMI) 1640 medium (Life Technologies) with 10% of fetal bovine serum (FBS, Sigma) for 45 min at room temperature with rotation. After incubation, the mixture was layered over cushion oil, composed by 85% silicone (SERVA) and 15% nujol (Thermo Fisher), and centrifuged to collect the RBCs. Bound proteins to the RBCs were eluted with 300 mM sodium chloride (NaCl), separated by SDS-PAGE and detected by Western blotting.

### DARC-binding assay and binding inhibition assay

The DARC-binding and binding inhibition assays were performed as described elsewhere^20^. Recombinant nDARC(Y)-Fc or nDARC(F)-Fc proteins (1 μg/ml) were coated on to Nunc MaxiSorp ELISA plates (Thermo Fisher) overnight at 4 °C in carbonate-bicarbonate buffer (capsules, Sigma). Next day, the plate was washed 3 times with PBS 0.05%Tween (PBS/T) and blocked for 2 h at 37 °C using 200 μl of 2% non-fat milk PBS/T. Recombinant PvDBPII SalI or PvW1 (0.8–25 ng/ml) were added in duplicates to DARC-coated plates. PvDBPII bound to DARC was probed with anti-PvDBPII polyclonal rabbit sera (dilution 1:10,000) at 37 °C for 1 h and detected with peroxidase-conjugated anti-rabbit IgG secondary antibody (dilution 1:10,000, Sigma) at 37 °C for 1 h. The assay was developed at room temperature using 100 μl of the two-component chromogenic substrate for peroxidase detection, TMB (3,3′,5,5′-tetramethylbenzidine, Life Sciences), for 5 min. The reaction was stopped with 100 μl of 1 M phosphoric acid (H_3_PO_4_). The optical density was immediately measured at a wavelength of 450 nm (OD_450_).

For the binding inhibition assay, PvDBPII SalI or PvW1 (0.8–25 ng/ml) were added on pre-blocked nDARC(Y)-Fc coated plates and used to generate a PvDBPII standard curve. Serum samples were analyzed at dilutions of 1:100 to 1:24,300. Each serum dilution was incubated with 25 ng/ml of PvDBPII SalI or PvW1 at 37 °C for 30 min. The reaction mixture was added to DARC-coated plates in duplicates and incubated at 37 °C for 1 h. PvDBPII bound to DARC was detected as described above. The amount of bound PvDBPII was estimated by converting OD_450_ values to protein concentrations using a four-parameter logistic model fitting the PvDPBII standard curve. The interpolated protein concentration values were used to calculate percent (%) binding for each serum sample dilution. Then, the % binding inhibition for each serum dilution was calculated as follows: %Binding inhibition = 100 − % Binding. The plot of % Binding Inhibition versus serum dilution was used to find the serum dilution at which 50% binding inhibition (IC_50_) is achieved for each serum sample.

### PvDBPII binding to DARC by biolayer interferometry (BLI)

Binding of PvDBPII with DARC was analyzed by the technique of biolayer interferometry (BLI) on an Octet RED 384 instrument (Fortebio). All measurements were made at 25 °C in standard Greiner black 96-well microtiter plates in a volume of 120 μl/well with shaking at 1000 rounds per minute (rpm). A buffer consisting of PBS with 1 mg/ml bovine serum albumin (BSA) was used as control, for baseline/dissociation steps and to dilute recombinant proteins. PvDBPII SalI and PvW1 were diluted to concentrations ranging from 1 to 120 nM. Anti-human IgG Fc Capture (AHC) Biosensors (Sartorius) were hydrated for 10 min in PBS and regenerated (3 cycles, 30 s each) with 10 mM glycine pH 1.5. The recombinant nDARC(Y)-Fc protein was immobilized via its Fc at 20 μg/ml for 600 s. Reference biosensors were prepared by loading human IgG1 Fc protein (Thermo Fisher) at 5 μg/ml for 600 s on AHC Biosensors. Loaded biosensors were assayed for binding to PvDBPII SalI or PvW1 with the following sequence of steps: baseline (60 s in buffer), association (with recombinant PvDBPII dilutions for 3600 s) and dissociation (600 s in buffer). Wells containing only buffer were assigned as reference sample wells. Signals from reference biosensors and reference sample wells were subtracted to determine the specific PvDBPII-DARC binding signals. Affinity constants (K_D_) were determined by fitting the association/dissociation profiles using a 1:2 binding model and performing a Steady-state analysis using the Octet Data Analysis HT software version 11 (Fortebio). Two independent experiments were performed and averaged to report the K_D_ of each PvDBPII protein with standard deviations (SD).

### ELISA

Nunc MaxiSorp ELISA plates were coated with 100 μl of recombinant PvDBPII SalI or PvW1 proteins at 1 μg/ml in carbonate-bicarbonate buffer overnight at 4 °C. The next day, plates were washed three times with PBS/T and blocked with 5% non-fat milk PBS/T for 1 h at 37 °C. Mouse sera were diluted in 2.5% non-fat milk PBS/T (initial dilution 1:2000) and 100 μl per well was added in duplicates and incubated for 1 h at 37 °C. After washing with PBS/T, bound antibodies were detected by adding 100 μl of horseradish peroxidase-conjugated anti-mouse IgG antibody (dilution 1:3000, Promega) and incubating for 1 h at 37 °C. The assay was developed as described above in the DARC-Binding Assay protocol. OD_450_ values and serum dilutions were used to fit a four-parameter logistic model. The effective serum concentration at which 50% binding was observed (EC_50_) is reported.

### Avidity

PvDBPII SalI and PvW1 were coated on ELISA plates as described above in the ELISA protocol. PvDBPII coated wells were incubated with sera samples diluted to give an OD_450_ of ~ 2.0 in duplicates. After sample incubation, descending concentrations of the chaotropic agent sodium thiocyanate (NaSCN, Sigma) (7 M to 0 M in PBS) were added (100 μl) and incubated for 15 min at room temperature. Plates were washed with PBS/T and reaction was developed as per the DARC-Binding Assay protocol described above. OD_450_ values were plotted versus NaSCN concentration and fitted in a four-parameter logistic model. The NaSCN concentration that resulted in a 50% reduction of the OD_450_ was used as a measure of avidity (IC_50_).

### Isotyping

The assay was performed as described for the ELISA protocol above except that mouse isotypes and subclasses were detected using the ISO2-1KT Mouse Monoclonal Antibody Isotyping Reagents (Sigma). Briefly, ELISA plates pre-coated with PvDBPII SalI were incubated with serum samples diluted at 1:400, 1:4000 and 1:40,000 in duplicates. After washing wells with PBS/T, the goat antibodies to mouse IgG1, IgG2a, IgG2b, IgG3, IgA and IgM were diluted at 1:1000 and 100 μl and added to the ELISA wells. After 1 h incubation at 37 °C, plates were washed and HRP-conjugated anti-goat antibody (Promega) was added (100 μl at dilution 1:5000). After a 1 h incubation at 37 °C, the reaction was developed as described above in the DARC-Binding Assay protocol. The OD_450_ of each sample was used to evaluate the IgG subclass, IgA or IgM isotype.

### Immunofluorescence assay (IFA)

A Clinical isolate collected from a *P. vivax* malaria patient in Cambodia during field surveys by Institut Pasteur du Cambodge was used for IFA and PvDBPII sequence was determined by Sanger sequencing as described before^21^. An informed written consent was obtained from patient prior to enrollment. All procedures were carried out in strict accordance with relevant guidelines and regulations. The *P. vivax* clinical isolate was matured to schizont stage in culture. Schizonts were purified on Percoll and used to prepare slides for use in immunofluorescence assays. Slides were fixed and frozen at − 70 °C in presence of desiccant. Frozen and fixed slides of *P. vivax* schizonts were thawed at room temperature for 30 min. Slides were blocked with 5% BSA in PBS for 30 min at 37 °C. Slides were incubated with PvDBPII-immunized mouse sera diluted in 2.5% BSA at 1:500 for 30 min at 37 °C, followed by three washes with PBS. A mixture of Alexa Fluor 488-conjugated goat anti-mouse IgG (H+L) secondary antibodies (Molecular Probes) at 1:500 and Hoechst 33342 solution (Molecular Probes) at 1:20,000 was added and incubated for 30 min at 37 °C. After washing, slides were treated with anti-Fade (Molecular Probes) and visualized on Leica DM 5000B Microscope (Leica Microsystems).

### Statistical analysis

Data analysis was performed using GraphPad Prism version 9.3.1 (GraphPad Software Inc.). Affinity constants of DARC-binding to PvDBPII SalI and PvW1 were using the Mann–Whitney test. Pairwise comparisons with Bonferroni’s multiple comparisons tests were performed for the results obtained in the DARC-Binding Assay, ELISA, avidity and Binding Inhibition Assay. Correlations between avidity, antibody and inhibitory titres for PvDBPII SalI and PvW1 were determined by Spearman rank test. All statistical tests were two-sided and a p-value < 0.05 was considered significant.

### Ethics statement

Human peripheral blood samples were collected from healthy volunteers through the ICAReB platform (Clinical Investigation & Access to Research Bioresources) from the Center for Translational Science, Institut Pasteur^22^. All participants received an oral and written information about the research and gave written informed consent in the frame of the healthy volunteers Diagmicoll cohort (Clinical trials NCT 03912246) after approval of the CPP Ile-de-France I Ethics Committee (2009, April 30th) and CoSImmGEn cohort (Clinical trials NCT 03925272), after approval of the CPP Ile-de-France I Ethics Committee (2011, Jan 18th)). All procedures were carried out in strict accordance with relevant guidelines and regulations.

Animal studies were carried out in strict accordance with the recommendations in the Guide for the Care and Use of Laboratory Animals of the Institut Pasteur (http://webcampus.pasteur.fr/jcms/c_283578/procedures-approuvees-par-le-comite-d-ethique) and complied with the European Union guidelines for the handling of laboratory animals (http://ec.europa.eu/environment/chemicals/lab_animals/home_en.htm) and ARRIVE guidelines. The procedures used were approved by the Institut Pasteur animal care and use committee. Animal care and handling was approved by the Ministère de l’Enseignement Supérieur de la Recherche et de l’Innovation (Ref. APAFIS 8845-2017122117082418v3). All animal experiments were planned and executed in order to minimize animal suffering.

## Results

### PvDBPII SalI and PvW1 variants bind similarly to DARC

Both PvDBPII recombinant proteins were expressed in *E. coli* as previously described^9–12^. SDS-PAGE analysis showed that both recombinant PvDBPII SalI and PvW1 migrate with an apparent molecular weight of 39 kDa, as expected, and the monomeric PvDBPII band reveals a purity of more than 95% as comparison to the BSA standards (5 μg of monomeric PvDBPII SalI and PvW1 did not show other bands with similar intensity to the BSA standard of 0.25 μg, Supplementary Figs. S1A, S2B). Analysis on SDS-PAGE gels reveals that both PvDBPII SalI and PvW1 migrate with slightly different mobilities under non-reducing (NR) and reducing (R) conditions (Supplementary Figs. S1B, S2C). The shift in mobility indicates formation of disulfide bonds. PvDBPII SalI and PvW1 were both recognized by Western blotting using anti-6xHis tag antibodies, which recognized the C-terminal 6xHis tags on both parasite antigens (Supplementary Figs. S1B, S2C).

**Figure 1:**
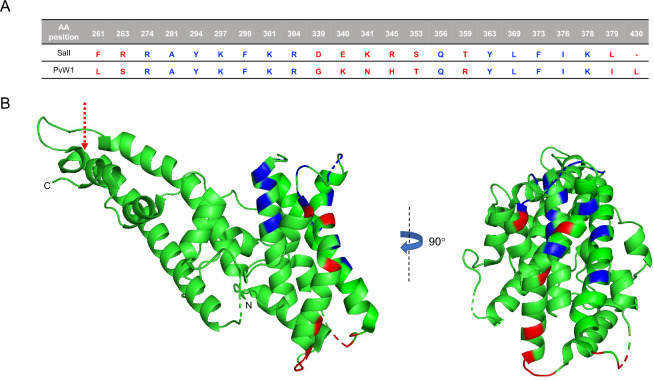
Polymorphisms and binding residues in the PvDBPII SalI and PvW1. (**A**) Binding residues that remain conserved (blue) and polymorphisms (red) between PvDBPII SalI and PvW1 are shown. (**B**) PvDBPII SalI structure (green) including the binding residues (blue) and the PvW1 polymorphisms (red) are shown. The leucine insertion between positions 429 and 430 in the SalI sequence is indicated with a dashed arrow. The amino and carboxyl termini of PvDBPII are indicated with N and C, respectively. Structures were obtained from the Protein Data Bank (PDB) structure 4NUV and modified using PyMOL software version 1.2.

**Figure 2:**
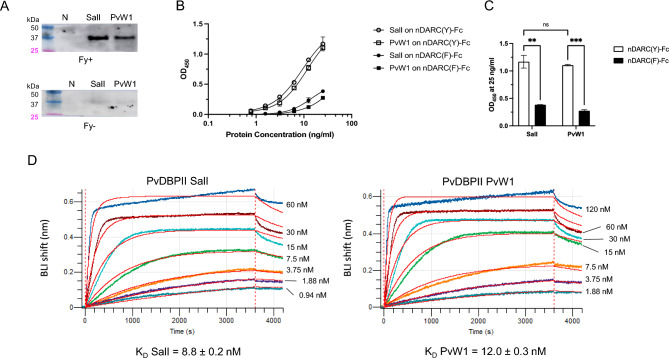
Binding of the recombinant PvDBPII variants SalI and PvW1 to DARC. (**A**) Binding to Duffy positive (Fy+) or Duffy negative (Fy−) erythrocytes of the recombinant PvDBPII variants SalI and PvW1 were detected by Western blotting. Erythrocyte Binding assay with no recombinant protein was used as a negative control (N). (**B,C**) Binding to nDARC(Y)-Fc and nDARC(F)-Fc of PvDBPII SalI and PvW1 at different concentrations (**B**) and at 25 ng/ml (**C**). Means and SD are shown. p values **p < 0.01, ***p < 0.001, pairwise comparisons with Bonferroni’s multiple comparison tests. (**D**) Binding kinetics of the PvDBPII SalI and PvW1 to nDARC(Y)-Fc by BLI. Different concentrations of PvDBPII domains ranging from 1 to 120 nM were tested to bind nDARC(Y)-Fc. The affinity constant, K_D_, was calculated by fitting the association/dissociation profiles with a 1:2 binding model and performing a Steady-state analysis. Two independent experiments were performed, and the K_D_ was averaged and reported for each PvDBPII protein ± SD.

The PvW1 sequence obtained from a Thai isolate^19^ contains 10 polymorphisms, including a leucine insertion between positions 429 and 430 of the SalI sequence (Fig. 1A). Mapping these polymorphisms on the PvDBPII structure obtained by Batchelor et al.^23^ shows that they do not correspond to the residues on PvDBPII that are essential for binding to DARC^21,24^ (Fig. 1B). Indeed, recombinant PvW1 binds to Duffy positive (Fy+) but not Duffy negative (Fy−) RBCs (Fig. 2A, Supplementary Fig. S2A). In addition, the binding of PvDBPII to DARC was shown to heavily rely on the sulfation of the DARC tyrosine 41 (Y41) residue^25^. A soluble DARC with sulphated Y41 is able to block the interaction of PvDBPII with RBCs^25^. In addition, when this tyrosine residue is substituted with a phenylalanine (absence of sulphated Y41) the DARC-PvDBPII interaction is strongly impaired^25^. As in the case of PvDBPII SalI, binding of PvDBPII PvW1 to DARC is also dependent on the sulfation of Y41 on DARC (Fig. 2B). Binding of recombinant PvDBPII SalI and PvW1 at the highest concentration tested (25 ng/ml) was significantly higher for nDARC(Y)-Fc compared to nDARC(F)-Fc but no differences in binding were observed between the two PvDBPII variants (Fig. 2C).

Finally, binding constants of PvDBPII SalI and PvW1 to nDARC(Y)-Fc were determined by BLI (Fig. 2D). Affinity constants for the interaction of PvDBPII SalI and PvW1 with nDARC(Y)-Fc were similar with no statistically significant difference in K_D_ values (K_D_ for SalI was 8.8 ± 0.2 nM and K_D_ for PvW1 was 12.0 ± 0.3 nM, Mann–Whitney test p = 0.33). These results show that PvDBPII SalI and PvW1 bind with similar binding affinities to DARC.

### Immunogenicity of PvDBPII SalI/Matrix-M and PvDBPII SalI/GLA-SE in mice

**Figure 3:**
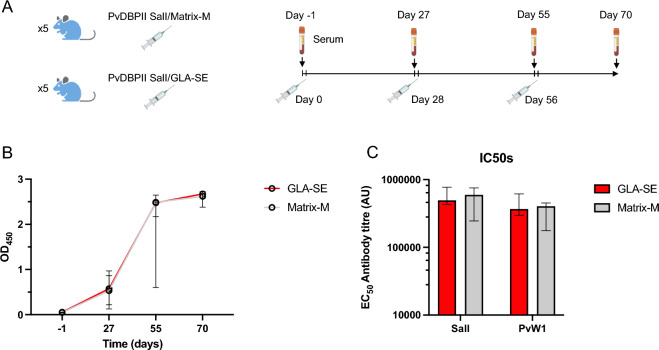
Anti-PvDBPII antibodies elicited in mice can recognize both SalI and PvW1. (**A**) Scheme of mice immunizations with PvDBPII. Five mice received PvDBPII SalI formulated with Matrix-M or GLA-SE at days 0, 28 and 56. Mice sera was collected at days − 1, 27, 55 and 70. Images were generated in online portal Biorender (**B**) Antibody kinetics measured by ELISA over the course of the immunizations. Median OD_450_ at dilution 1:50,000 of each mice group including range is shown. (**C**) Antibody titres reported as effective concentrations (EC_50_) that can recognize PvDBPII SalI and PvW1 in both mice groups. Median and range are shown.

Recombinant PvDBPII SalI formulated with Matrix-M and GLA-SE was used to immunize BALB/c mice as detailed in Fig. 3A. Five mice per group were immunized and sera were collected one day prior to each immunization. Final bleeds were collected 2 weeks after the final boost and serum samples at each time point were evaluated for presence of anti-PvDBPII IgGs by ELISA (Fig. 3B). The OD_450_ signals in both GLA-SE and Matrix-M groups increased after each immunization although the increase after the second boost did not reach significance (Fig. 3B). OD_450_ at day − 1 was less than 0.1 for all mice. Serum samples at day 70 were tested for recognition of both PvDBPII variants SalI and PvW1 (Fig. 3C). Both adjuvant groups showed similar recognition titers for both SalI and PvW1, indicating Matrix-M and GLA-SE elicited comparable PvDBPII-specific antibody responses and these antibodies can equally recognize both PvDBPII variants.

### Immunized sera recognize native PvDBP in *P. vivax* schizonts

**Figure 4:**
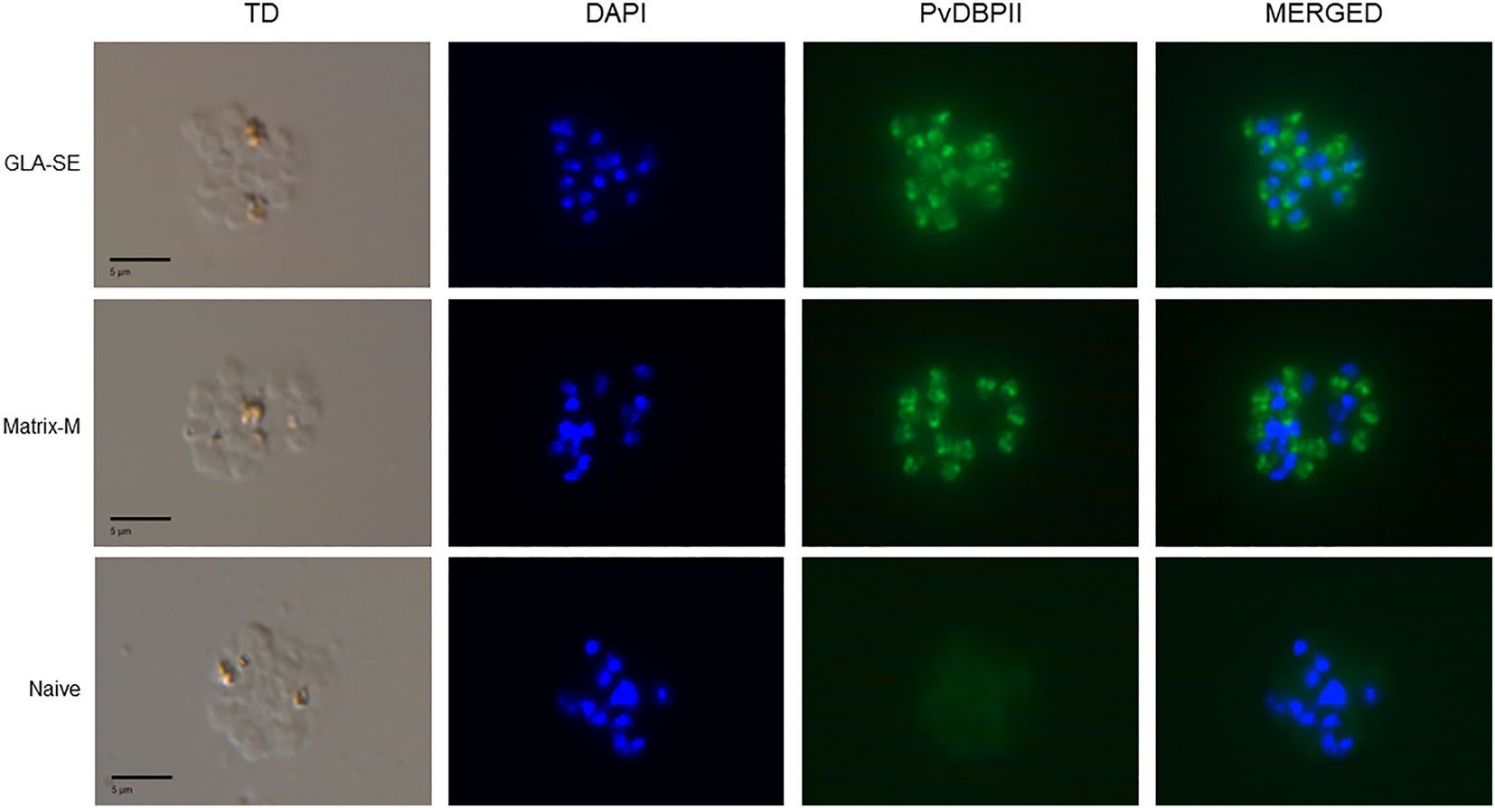
Reactivity of mouse sera with *P. vivax* schizonts from infected *P. vivax* malaria patients. Representative images of *P. vivax* schizonts incubated with sera from mice immunized with PvDBPII formulated with GLA-SE and mice immunized with PvDBPII formulated with Matrix-M showing apical staining of merozoites (green) in mature *P. vivax* schizonts compared to a pool of mice sera at day − 1 (Naive). This clinical isolate contains 3 mutations in the PvDBP RII sequence compared to Sal1 reference sequence: R263S, D339G and R345H.

Serum samples at day 70 were tested for recognition of native PvDBP in *P. vivax* parasites by IFA. All five mice in both adjuvant groups showed apical staining corresponding to the known localization of PvDBP in micronemes (Fig. 4). Incubation of *P. vivax* schizonts with sera from mice immunized with Matrix-M and GLA-SE formulations of PvDBPII showed similar patterns and intensity of fluorescence signal. A pool with mouse sera at day − 1 (Naive) showed no reactivity.

### Functional analysis of the PvDBPII antibody responses

**Figure 5:**
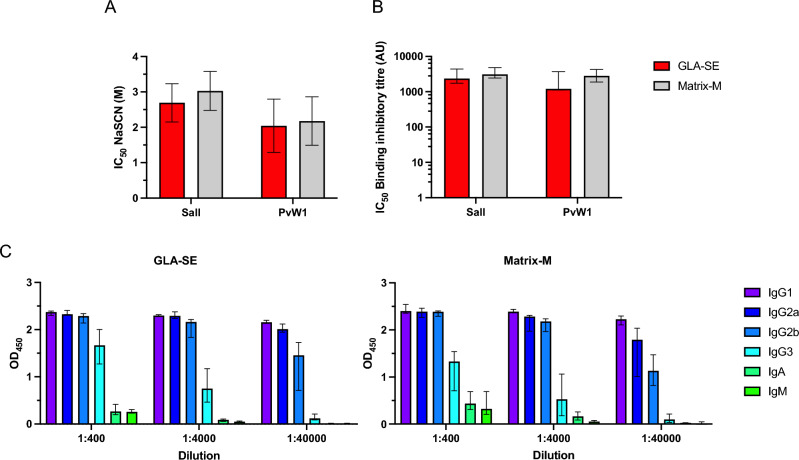
Functional characterization of anti-PvDBPII antibodies. (**A**) Avidity of PvDBPII-specific antibodies at day 70 elicited in GLA-SE and Matrix-M adjuvant groups to PvDBPII SalI and PvW1. (**B**) Binding inhibitory titres at day 70 that block interaction between DARC and PvDBPII SalI and PvW1. (**C**) Isotypes of antibodies raised against PvDBPII for each adjuvant group. (**A–C**) Median and range are shown.

**Figure 6:**
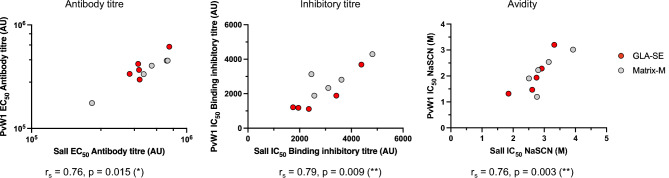
Correlations of functional properties between PvDBPII SalI and PvW1. The PvW1-specific antibody titres, binding inhibitory titres and avidity are associated with the PvDBPII SalI responses. Spearman’s rank correlation coefficient and p values are shown.

Next, anti-PvDBPII antibodies collected at day 70 from mice immunized with PvDBPII formulated with Matrix-M and GLA-SE were analyzed for avidity, isotyping and PvDBPII-DARC binding inhibition. Avidity was similar for both adjuvant groups with no statistically significant differences between the SalI and PvW1 variants or between adjuvants (Fig. 5A). Serum samples were also tested for inhibition of the PvDBPII-DARC interaction in a Binding Inhibition Assay. Both adjuvant groups elicited PvDBPII-DARC binding inhibitory antibody responses (Fig. 5B). No differences were found between the two adjuvant groups and DARC-binding was blocked at similar levels for both the PvDBPII SalI and PvW1 variants. These results demonstrate that immunization with PvDBPII formulated with both Matrix-M and GLA-SE can elicit binding inhibitory titres that can block DARC-binding of the homologous SalI domain as well as the heterologous PvW1 variant with similar efficiency. In addition, the avidity and binding inhibitory titres as well as the antibody titres positively correlate between the two PvDBPII variants (Fig. 6).

Finally, the profile of antibody responses was determined by isotyping. The following relative predominance for isotype/subclass was observed: IgG1 = IgG2a > IgG2b > IgG3 > IgA = IgM, in both adjuvant groups (Fig. 5C). Thus, we found that a similar antibody profile was obtained after immunization with both Matrix-M and GLA-SE formulations of PvDBPII.

## Discussion

Adjuvants and formulations are usually tested in pre-clinical and early clinical studies during vaccine development. The leading *P. vivax* vaccine candidate PvDBPII formulated with GLA-SE was shown to be safe and immunogenic in a dose-escalating Phase 1 clinical trial^14^. Concurrently, the saponin-based adjuvant, Matrix-M, was shown to confer excellent safety and immunogenicity profiles in pre-clinical and clinical studies with other malaria vaccines. A recombinant *P. falciparum* malaria vaccine R21 based on the fusion of the PfCSP antigen with HBsAg, the surface antigen of hepatitis B virus formulated with Matrix-M yielded promising levels of efficacy against *P. falciparum* malaria in challenge trials followed by efficacy trials in children in the field^26^. A similar vaccine for *P. vivax* malaria, Rv21, composed of PvCSP fused to HBsAg elicited protection in pre-clinical mouse models against challenge with transgenic *P. berghei* sporozoites expressing PvCSP^18^. Here, we explored the feasibility of formulating PvDBPII SalI with Matrix-M in a pre-clinical mouse model and compared the immunogenicity with GLA-SE formulation. We have examined the ability of antibodies elicited by immunization with Matrix-M and GLA-SE formulations of PvDBPII SalI to inhibit receptor-binding by both the homologous SalI and heterologous PvW1 allele from the *P. vivax* isolate used for blood stage challenge trials.

GLA-SE is a synthetic Toll-like receptor 4 (TLR4) agonist, which induces production of pro-inflammatory cytokines and stimulates antigen presenting cells like dendritic cells^27,28^. In the case of Matrix-M, no specific receptor—including TLR—has been identified. Formulation of polysaccharide antigen with another QS extract, QS-21, elicited high titre antibodies in transgenic mice defective in TLR4 signaling^29^, suggesting that QS extracts may have unknown mechanisms to activate innate immune responses. Matrix-M, as a formulated saponin extract like QS-21, may similarly have alternate mechanisms to activate host innate immunity and thus support high titre antibody responses. Even so, important similarities between both adjuvants have been found such as the recruitment to the draining lymph nodes of MHC class II-expressing dendritic cells that upregulate co-stimulatory molecules^30,31^. Independent of the different immune receptors triggered by GLA-SE or Matrix-M, we have observed a strong similarity in the antibody responses elicited against PvDBPII. After PvDBPII immunization with either one of the adjuvants, antibody levels rose equally during the immunization course reaching similar EC_50_ values at day 70. The PvDBPII-specific antibodies elicited by both GLA-SE and Matrix-M formulations recognize native PvDBP in *P. vivax* schizonts. Furthermore, these PvDBPII-specific antibodies showed comparable avidity and IC_50_ values for inhibition of PvDBPII-DARC-binding. Though no cellular immune responses were analyzed in this study, the antibody isotypes and subclasses had the same predominance in both mice groups, which indicates that both adjuvants are likely to induce similar Fc-related cellular processes. From our results, we conclude that both adjuvants elicit comparable antibody responses after PvDBPII vaccination in mice. Thus, PvDBPII formulated with Matrix-M may exhibit similar immunogenicity as observed previously with the GLA-SE formulation in humans.

In this study, we evaluated antibody responses elicited by immunization with PvDBPII SalI for recognition and binding inhibition by the highly divergent PvDBPII variant PvW1^19^. Recombinant PvW1 showed binding specificity to Fy+ erythrocytes and Y41-sulfated recombinant DARC as previously observed^9,25^. In addition, the affinity constants, K_D_, for the binding of recombinant DARC to PvDBPII SalI and PvW1 were similar. The polymorphisms present in PvW1 from the *P. vivax* isolate used for blood stage challenge are frequently found in multiple Thai *P. vivax* isolates^32^. As per our results, PvDBPII immunization in mice with GLA-SE and Matrix-M generates antibodies that are able to recognize and inhibit DARC-binding by both SalI and PvW1 with similar efficiency. No statistically significant differences were observed in ELISA recognition titre, binding inhibitory titre or avidity of anti-PvDBPII antibodies for both the SalI and PvW1 alleles. In addition, significant positive correlations were found in these 3 measurements for the two PvDBPII variants, suggesting that immunization with PvDBPII SalI can elicit strain-transcending antibodies. Interestingly, the PvW1 polymorphisms do not correspond to essential DARC-binding residues indicating that antibodies targeting the binding residues should be cross-reactive and able to neutralize diverse variants. Further studies are needed to identify the epitopes on PvDBPII recognized by inhibitory antibodies.

In summary, we have shown that PvDBPII formulated with GLA-SE and Matrix-M elicit similar antibody responses in mice. These results suggest that Matrix-M could be used for formulation of PvDBPII. Importantly, the DARC-binding inhibition of PvW1 also indicates that the PvDBPII-specific antibodies can potentially block invasion of the *P. vivax* strain PvW1 that is used for blood stage challenge trials with *P. vivax*^19^. Indeed, PvDBPII/Matrix-M was tested for efficacy in a challenge trial and was found to elicit immune responses that reduced parasite multiplication rate of the heterologous challenge strain PvW1 by 53% compared to unvaccinated controls^33^. This is the highest reduction in parasite multiplication rate achieved by a blood-stage malaria vaccine^34–39^. The combination of multiple antigens is an attractive approach to increase vaccine efficacy. As shown in the rodent model of infection with *P. yoelii*, the co-administration of the blood-stage antigen *P. yoelii* merozoite surface antigen 1 (PyMSP_1_) and the pre-erythrocytic antigen PyCSP results in an increased delay in patent parasitemia after sporozoite challenge compared to immunization with PyCSP alone^40^. Similarly, combination of a PvCSP based vaccine and PvDBPII could synergistically improve vaccine efficacy in humans.

### Supplementary Information

Supplementary Figures.

## Data Availability

The datasets generated during and/or analysed during the current study are available from the corresponding author on reasonable request.
