# Supplementary material for: Immunogenicity of a Plasmodium vivax vaccine based on the duffy binding protein formulated using adjuvants compatible for use in humans
Source: Sci Rep. 2023 Aug 25;13:13904. doi: 10.1038/s41598-023-40043-6 (PMC10457348; doi:10.1038/s41598-023-40043-6)
Supplement: Supplementary file 1 — Supplementary Figures. [file 41598_2023_40043_MOESM1_ESM.docx]

**Immunogenicity of a *Plasmodium vivax* Vaccine Based on the Duffy Binding Protein Formulated Using Adjuvants Compatible for Use in Humans**

Francisco J. Martinez^1^, Micheline Guillotte-Blisnick^1^, Christèle Huon^1^, Patrick England^2^, Jean Popovici^3^, Hélène Laude^4^, Laurence Arowas^4^, Marie-Noëlle Ungeheuer^4^, Jenny M. Reimer^5^, Darrick Carter^6,7^, Steve Reed^6^, Paushali Mukherjee^8^, Virander S. Chauhan^9^ and Chetan E. Chitnis^1,^*

^1^Unité de Biologie de Plasmodium et Vaccins, Institut Pasteur, Université Paris Cité, Paris, France.

^2^Plate-forme de Biophysique Moléculaire, Institut Pasteur, CNRS UMR 3528, Université Paris Cité, Paris, France.

^3^Malaria Research Unit, Institut Pasteur du Cambodge, Phnom Penh, Cambodia.

^4^Investigational Clinical Service and Access to Research Bio-resources (ICAReB), Institut Pasteur, Paris, France.

^5^Novavax AB; Kungsgatan 109, SE-753 18, Uppsala, Sweden.

^6^HDT Bio, Seattle, WA.

^7^PAI Life Sciences Inc., Seattle, WA.

^8^Multi-Vaccines Development Program, ICGEB Campus, New Delhi, India.

^9^International Centre for Genetic Engineering and Biotechnology (ICGEB), New Delhi, India.

*Address correspondence to: Prof. Chetan E. Chitnis, Institut Pasteur, 25-28 Rue du Dr. Roux, Paris 75015, France ; Tel/FAX: +33 1 44 38 94 28; E-mail: chetan.chitnis@pasteur.fr

**Supplementary Figures**


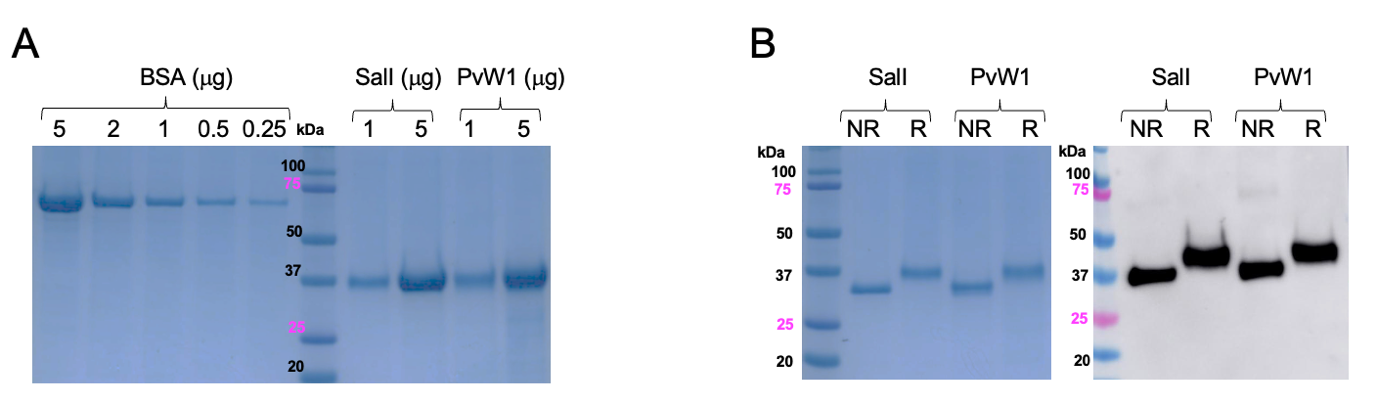


**Supplementary Figure S1. Expression of recombinant PvDBPII SalI and PvW1.** (**A**) Purity of PvDBPII SalI and PvW1 on SDS-PAGE gels stained with Coomasie Blue. Known amounts of bovine serum albumin (BSA) were used as control. (**B**) Migration profiles of PvDBPII SalI and PvW1 under non-reducing (NR) and reducing (R) conditions detected by Coomasie staining and Western blotting with anti-6xHis antibodies. Uncropped gels and blots are shown in Supplementary Figure S2A and B.


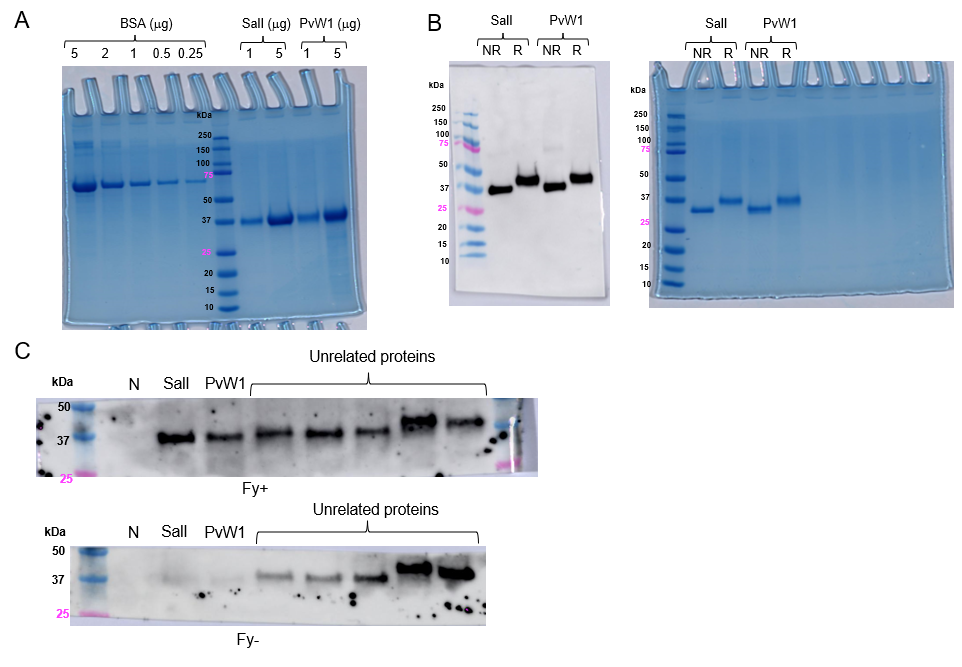


**Supplementary Figure S2. Original electrophoretic gels and Western blots.** (**A**) Coomasie gel for Supplementary Figure S1A showing purity of PvDBPII SalI and PvW1 recombinant domains. (**B**) Western blot and Coomasie gel for Supplementary Figure S1B indicating the migration profiles of PvDBPII SalI and PvW1 under non-reducing (NR) and reducing (R) conditions. (**C**) Western blot for Figure 2A showing binding of the recombinant PvDBPII variants SalI and PvW1 to Duffy positive (Fy+) or Duffy negative (Fy-) erythrocytes. Erythrocyte Binding assay with no recombinant protein was used as a negative control (N). Bands of proteins unrelated to the experiment are indicated.
